# A Rapid Response Electrochemical Biosensor for Detecting Thc In Saliva

**DOI:** 10.1038/s41598-019-49185-y

**Published:** 2019-09-03

**Authors:** Hunter Stevenson, Amanda Bacon, Kathleen Mary Joseph, Wilma Ruth Wanjiku Gwandaru, Ashlesha Bhide, Devangsingh Sankhala, Vikram Narayanan Dhamu, Shalini Prasad

**Affiliations:** Department of Biomedical Engineering, University of Texas at Dallas, 800W Campbell Rd., Richardson, TX 75080 USA

**Keywords:** Biosensors, Nanofabrication and nanopatterning

## Abstract

Marijuana is listed as a Schedule I substance under the American Controlled Substances Act of 1970. As more U.S. states and countries beyond the U.S. seek legalization, demands grow for identifying individuals driving under the influence (DUI) of marijuana. Currently no roadside DUI test exists for determining marijuana impairment, thus the merit lies in detecting the primary and the most sought psychoactive compound tetrahydrocannabinol (THC) in marijuana. Salivary THC levels are correlated to blood THC levels making it a non-invasive medium for rapid THC testing. Affinity biosensing is leveraged for THC biomarker detection through the chemical reaction between target THC and THC specific antibody to a measure signal output related to the concentration of the targeted biomarker. Here, we propose a novel, rapid, electrochemical biosensor for the detection of THC in saliva as a marijuana roadside DUI test with a lower detection limit of 100 pg/ml and a dynamic range of 100 pg/ml – 100 ng/ml in human saliva. The developed biosensor is the first of its kind to utilize affinity-based detection through impedimetric measurements with a rapid detection time of less than a minute. Fourier transform infrared spectroscopy analysis confirmed the successful immobilization of the THC immobilization assay on the biosensing platform. Zeta potential studies provided information regarding the stability and the electrochemical behavior of THC immunoassay in varying salivary pH buffers. We have demonstrated stable, dose dependent biosensing in varying salivary pH’s. A binary classification system demonstrating a high general performance (AUC = 0.95) was employed to predict the presence of THC in human saliva. The biosensor on integration with low-power electronics and a portable saliva swab serves as a roadside DUI hand-held platform for rapid identification of THC in saliva samples obtained from human subjects.

## Introduction

Marijuana is commonly used as a recreational drug due to its stimulant and euphoric effects. Tetrahydrocannabinol (THC) is the primary psychoactive compound in marijuana, which acts on the endocannabinoid system within the central nervous system^1,2^. Its agonism on the cannabinoid receptors alters the concentrations of various neurotransmitters (e.g. dopamine and norepinephrine) which are closely associated with THC’s effect on mood and conscious perception^2^. Effects of consuming marijuana include but are not limited to: euphoria, stress reduction, increased humor and music appreciation, metacognition, and creativity^3^. Anxiety and or panic attacks are the most common side effects of smoking marijuana when consumed at doses exceeding the psychotropic threshold^4^. When smoking marijuana, these effects can wholly manifest within a few minutes and last 1–3 hours^4^. Whereas oral consumption of marijuana drastically prolongs the time for effects to arise and subsequently diminish due to the slower adsorption through the gut^4^. THC is a lipophilic molecule and will bind nonspecifically to other fat-containing body parts such as adipose tissue^3^. Because it can store in fat tissue, THC yield a longer elimination half-life (EHL) relative to other recreational drugs. Furthermore, the EHL is also dependent on metabolism, and quantity/frequency of use. In fact, the redistribution of THC from tissue to blood is the rate-limiting step in its metabolic pathway^3^.

Regular use of marijuana shows an increased risk of addiction and the use of other illicit drugs^5^. There is evidence that suggests long-term use of marijuana may lead to addiction in approximately 9% of those who use marijuana^5,6^. Certain groups are at an elevated risk for addiction; those who start using marijuana as teenagers have a 1 in 6 chance of developing marijuana dependency and daily user dependency rates are between 25 to 50%^5^. The *Diagnostic and Statistical Manual of Mental Disorders*, 4^th^ edition (DSM-IV) demonstrates that of the 5.1 million people that meet the criteria for dependency on any illicit drug, over half of these cases are due to marijuana^6^. In addition to risks of addiction, consumption of marijuana can impair neural connectivity and lead to learning and memory loss, diminished alertness and self-awareness, as well as degradation of habit and routine management^7^.

Marijuana is among the most commonly detected drugs in random roadside screenings^5^. Increased blood THC concentrations and recent smoking (within 2 hours) are strongly associated with higher crash and culpability risks^8^. These risks arise because THC interferes with visual and auditory perception as well as key psychomotor abilities^4^. A 2012 review of marijuana consumption found that driving under the influence of marijuana correlates to nearly a 2x increased risk of motor vehicle collisions, especially in fatal collisions^9^. Another study found that marijuana was among the most frequently detected drug among motor vehicle crash victims admitted to regional Level-I trauma centers within the US^10^. Onsite, roadside testing for THC is routinely performed in urine despite urine being a poor indicator of recent marijuana use. Instead, there is a push for law enforcement to utilize tests using saliva as the detection medium, as the salivary THC profile closely correlates to levels in blood^11^.

Urine and blood are the most common bodily fluid matrices used to test for marijuana use. However, saliva, sweat, and hair have also been explored for alternative, less invasive tests^12^. The window of detection and cut-off limit for each of these matrices vary significantly. THC can be detected in blood for up to 5 hours with a cutoff limit of 10 ng/mL, in urine for up to 95 days with a cutoff limit of 15 ng/mL, and in saliva for up to 34 hours with a 0.5 ng/mL limit^13,14^. However, these values are based on only a few seminal studies as approval for testing illicit products like marijuana are difficult to get approval for. The lack of reliable tests and cut-off limits for intoxication are common topics amongst debates to repeal marijuana prohibition. Furthermore, variable EHL and metabolism make setting cutoff concentrations for urine tests more difficult as the window of detection can vary drastically from subject to subject^15^. These large detection windows may be beneficial in the workplace where a zero-tolerance policy is in effect, but for roadside DUI testing a detection window should correlate closely to intoxication levels.

It is generally accepted that the presence of THC in oral fluid is an indicator of recent cannabis use^16^. When smoking marijuana, THC is deposited in the oral cavity through direct contact with oral mucosa; reuptake into saliva from blood also occurs but its effects are minimal^16^. Additionally, THC appears immediately within blood after the first inhalation and peaks within 8 minutes^13^. Despite that salivary THC levels have only an insignificant contribution directly from THC in blood, the time course in these two biological fluids follow very similar concentration profiles^16^. This finding suggests that saliva is a good indicator for active drug use.

Other reports of electrochemical detection of THC in saliva samples have been reported^12,16^. Here, we present an affinity-based biosensor that utilizes non-faradaic EIS to detect the presence of a BSA-THC hapten in human saliva samples. Affinity biosensors leverage biomarker detection through a recognition element that specifically targets the biomarker of interest. The chemical reaction between the target biomarker and the recognition element is then transduced to a measure signal output related to the concentration of the targeted biomarker. Affinity biosensors toward THC already exist as home-kit lateral^11^ flow assays, however to maintain an accurate reading these tests require: highly precise sample volumes, careful reagent handling by the user, and often relatively long wait times to results^11^. The electrochemical biosensor presented here could streamline the testing process, eliminate the need for any sample preparation and reduce the wait-time as the sensor does not rely on slow diffusion-limited processes. Additionally, the biosensor demonstrates early feasibility as a more sensitive and specific affinity-based test.

## Methods

### Sensor fabrication process

The biosensor was fabricated on PET (polyethylene terephthalate) substrates using the e-beam deposition technique. Electrodes were deposited using electron beam physical vapor deposition at a pressure of $5\ast {10}^{-6}\,torr$. The electrode patterns were deposited with 125 *nm* Au onto the precleaned PET substrate. After fabrication, the surface of the gold electrodes and the PET substrate were rinsed with 70% Isopropyl Alcohol (IPA), and distilled water (DI water) to remove organic residues. The electrodes were then dried with inert N_2_ gas to prepare the surface for self-assembly of the immunoassay. Construction of an immunoassay first chemisorbs a linker molecule to the electrode surface then binds a capture antibody for subsequent detection of a dynamic range of the THC-BSA hapten. The linker molecule specific to our biosensor is the amine-reactive crosslinker dithiobis (succinimidyl propionate) (DSP). DSP has an *N-*hydroxysuccinimide (NHS) ester that is highly reactive with the primary amines which are abundant in antibodies and a cleavable disulfide bond in the molecule’s spacer arm that interacts with the electrode surface via gold-thiol interaction^17^. DSP forms highly ordered self-assembled monolayers (SAM)^18^ which immobilize the anti-THC capture antibodies. The THC-BSA hapten possess high affinity toward the anti-THC antibody allowing for rapid binding interactions.

### Zeta potential evaluation as a function of pH for the THC-BSA hapten

A Malvern ZetaSizer Nano ZS was used to determine the zeta potential of the THC-BSA hapten as a function of pH. Samples of 0.05 g/L THC-BSA were prepared in 0.001 M KCl after titrating to varying pH by adding either 0.1 M HCl or 0.1 M NaOH. Samples were loaded into the folded capillary cell using a diffusion barrier technique^19^. First, 700 µL of the 0.001 M KCl was loaded into the DTS1070 capillary tube, then 100 µL of 0.05 g/L THC-BSA at matching pH was added to the bottom of the capillary cell using a gel-loading pipette. This diffusion barrier suspends the sample at the bottom of the cuvette, separating the THC-BSA hapten from the electrode protecting the molecule from damage by contact with the electrode. The zeta potential was measured using the electrophoretic mobility technique, which measures the velocity of the particles under the influence of an electric field, and then calculates zeta potential via the Smoluchowski equation given in Eq. 1:

1 $$\mu =\frac{{\epsilon }\zeta }{{\rm{\eta }}}$$

where $\,\mu$ represents the electrophoretic mobility, ${\epsilon }$ is the dielectric constant of the KCl dispersant, $\zeta$ is the zeta potential, and ${\rm{\eta }}$ is the viscosity of KCl dispersant (measured using a viscometer).

### Evaluation of dose-dependent response via electrochemical impedance spectroscopy

Electrochemical Impedance Spectroscopy (EIS) was employed as rapid, label-free approach to study the affinity-based detection of the THC-BSA hapten. The binding of the THC-BSA hapten to the anti-THC antibody conjugated to the sensor surface was analyzed by non-faradaic changes in the dielectric properties at the electrode-electrolyte interface. A Gamry Reference 600 potentiostat was used to measure the EIS response. An input 10 mV sinusoidal voltage with a DC bias of −0.3 V vs. Ref was applied at the working electrode, and then scanned across a frequency range of 1 Hz – 1 MHz. The measurement time is a function of the period of the frequencies used for the EIS scan. One sweep from 1 Hz to 1 MHz can be done in approximately 1 minute. The resultant current response was measured, and the complex impedance was calculated as the ratio of the input voltage to the output current.

**Figure 1:**
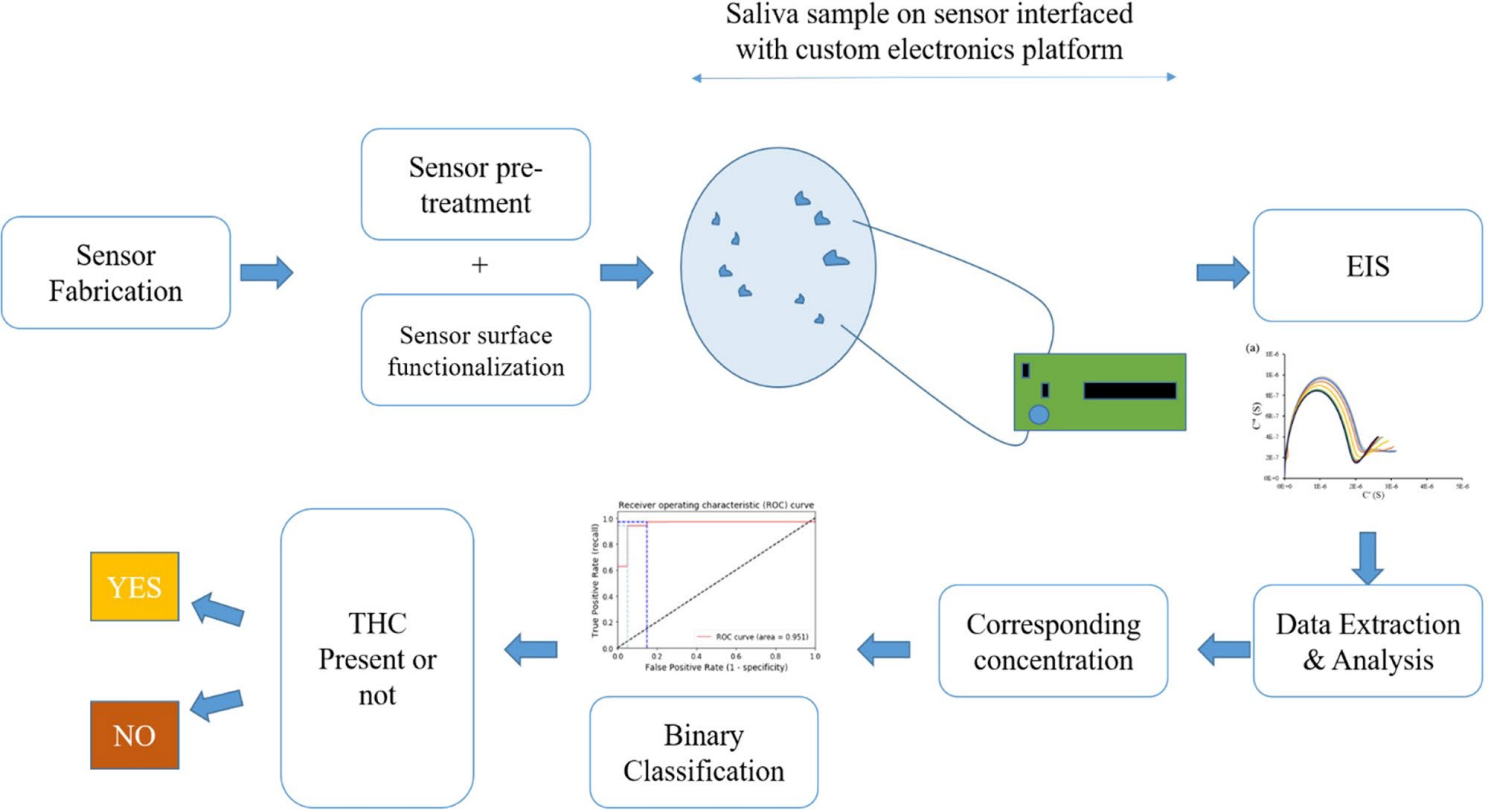
Schematic outlining the principal of operation of the THC biosensor.

EIS experiments were carried out first by diluting the THC-BSA hapten in 1x PBS to obtain concentrations between 100 pg/mL to 10 μg/mL to establish the baseline electrochemical interaction of the THC-BSA hapten with the electrode. After establishing the response in 1x PBS, the THC-BSA hapten was diluted in synthetic saliva at pH 4 and pH 6 to determine how pH affects the sensor’s dynamic response. Similarly, calibration dose response curve was established by spiking human saliva obtained commercially with THC concentrations between 100 pg/mL to 10 µg/mL. After assessing the sensor response in synthetic saliva, the sensor was tested on human saliva samples collected from human subjects to assess its performance as a salivary DUI dataset. All saliva samples were collected in accordance with the relevant guidelines and regulations through approval by the University of Texas at Dallas Institutional Review Board. Informed consent was obtained from all participants (all participants 18 years or older) before saliva donation. EIS measurements were taken again in response to incubating human saliva spiked with varying doses of THC (100 pg/mL, 10 ng/mL, & 100 ng/mL) after a 15-minute incubation on the sensor. A detailed schematic outlining the principal of operation of the THC biosensor is shown in Fig. 1.

### Optimal operating frequency selection for electrochemical impedance spectroscopy experiments

When interpreting non-faradaic EIS data, many researchers self-select the frequency range for their analysis based on assumptions about the chemical processes dominant at these frequencies^20^. This decision is largely dependent on the resistive and capacitive nature of the electrode, electrolyte, and biomarker of interest. Thus, some initial knowledge of the system characteristics should be understood before attempting to extract information. Researchers typically identify the optimal frequency for sensor operation by finding the frequency with a maximal signal-to-noise ratio (SNR) between their test samples and control (no analytes present)^20^. This optimal frequency is selected where either capacitive or resistive effects are dominant. Thus, biasing their analysis to one aspect of the electrochemical response and ignoring the remainder of the spectrum. While this is a widely accepted practice, the presence of the analyte may alter low frequency double layer capacitance (or charge transfer resistance) and high frequency solution resistance simultaneously.

A method of down-selecting the optimal frequencies is employed through a systematic, unbiased approach of feature selection methods developed for reducing data dimensionality. A Fast Correlation Based Filter (FCBF)^21,22^ was used to identify which combination of features contain the maximum discrimination information about the output. Using FCBF, features are deemed irrelevant only if they are conditionally independent of the different classes to predict on. FCBS has been demonstrated to improve learning algorithm speed and accuracy over other commonly employed filter methods. By removing irrelevant or highly correlated frequencies from the spectrum noise and redundancy are reduced which improves the generalization of the algorithm’s prediction power on new datasets.

Additionally, by removing unwanted data, the algorithm’s learning rate is dramatically improved, allowing for faster training and deployment^22,23^. FCBF was employed to remove features that that are poor predictors of the output class or highly correlated to ‘better’ input features. The goal of the FCBF algorithm was to identify the best feature(s) from 1) the raw impedance dataset, 2) the dataset normalized to the antibody, and 3) the dataset normalized to the synthetic saliva, while ensuring that each of these three features are not correlated to one-another.

### Binary classification of *THC*+/− human saliva samples

Here four binary classification algorithms were optimized to separate the $THC+$ inputs (any saliva sample containing >100 pg/mL THC) from the $THC-$ inputs (samples containing no THC). Saliva samples collected from human subjects spiked without and with varying doses of THC (100 pg/mL, 10 ng/mL, & 100 ng/mL) were utilized for the classification studies. Two Logistic Regression^24^ models – one without and one with K-folds cross-validation^25^ – and two Support Vector machines^26^ (SVM) – one with a linear kernel and one with a radial bias kernel^26^ – were developed. For each of the models, the percentage of $THC+$ samples that were correctly identified were calculated as the True Positive Rate (TPR), while the percentage of $THC+$ samples that were incorrectly classified referred to the False Positive Rate (FPR).

Receiver Operating Characteristic (ROC) curves were plotted with TPR, or sensitivity, on the y-axis and FPR on the x-axis^27^. The Area Under the ROC Curve (AUC) provides the aggregate performance for the model’s classification ability by exploring all possible threshold parameters. Because the AUC is classification-threshold-invariant, it indicates the quality of the classifier by examining the overall separability between the two classes^27^. ROC curves were evaluated based on the algorithm’s prediction power on unseen data points. For the first Logistic Regression model presented, a test was generated by randomly sampling 20% of the measurements from the dataset to hold out during the training phase (aka an 80/20 train/test split). 92 samples were used in the control group and 180 samples in the experimental group. To improve on the generalizability of the classification algorithms, K-folds cross-validation was implemented using K = 10 folds for the remaining three algorithms. Here, the dataset is divided into 10 equal partitions, and each partition is iteratively used as the test set while the remaining data is used to train the model. This allows every data point to be used in the training & testing process, leading to better performance by penalizing solutions that exhibit overfitting or high selection bias^25,27^.

One powerful advantage of SVM is that the algorithm can search in higher dimensional space to create non-linear decision boundaries using a kernel function^28^. The kernel function constructs a linear decision boundary in a high dimensional space then projects back down to the dimensions of the input space. Linear kernel SVMs assume the data are linearly separable in the input space (i.e. a line in 2D space, a plane in 3D space). The second SVM model used a radial bias function (rbf) kernel. The rbf kernel uses a gaussian distribution to describe the distance from the support vectors to the separating hyperplane, allowing non-linear, curved decision boundaries to be drawn.

## Results

### Characterization of electrode surface modifications by fourier-transform infrared spectroscopy (FTIR) analysis

Before implementing the biosensor toward THC detection, the chemisorption of the DSP cross-linker and anti-THC antibody on the gold electrode surface were validated using FTIR. Gold thin films were deposited on PET substrates using e-beam vapor deposition at parameters mimicking those used for sensor fabrication and used for FTIR analysis. Functionalization of the electrode surface was carried out as described in the previous section. Prior to FTIR measurements, each sample was rinsed thoroughly with DI water then dried with N_2_ air to rid the surface of any unbound material that may interfere with the analysis. The infrared spectra of surface modified samples were recorded with a Nicolet iS50 FTIR spectrometer. Absorbance spectral measurements were obtained with a scan resolution of 4 cm^−1^ for 64 scans in the spectral range of 4000 cm^−1^ to 600 cm^−1^. Absorption spectra was recorded for PET-gold surface conjugated to DSP molecules, and the gold surface with anti-THC antibodies linked via the DSP linker molecules.

**Figure 2:**
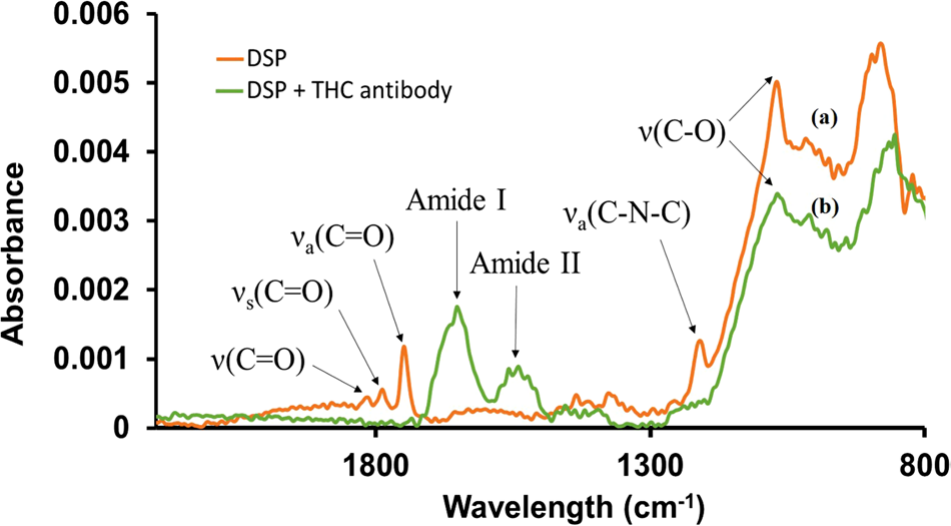
FTIR spectra of (**a**) DSP crosslinker immobilized on Au surface, (**b**) anti-THC antibody conjugated to DSP cross-linker.

The absorbance peaks observed in the DSP spectrum of Fig. 2 at 1786 cm^−1^ and 1749 cm^−1^ indicate the symmetric and asymmetric carbonyl stretches (respectively) of the NHS ester. Furthermore, the peak at 1212 cm^−1^ confirm the presence of the asymmetric C-N-C stretch of the NHS ester while the peak at 1069 cm^−1^ can be identified as the succinimide N-C-O stretch. Finally, the peak at 1170 cm^−1^ indicates the ester carbonyl stretch. The presence of these peaks is characteristic of a self-assembled monolayer of DSP, confirming the chemisorption of DSP to the gold surface^17^.

The reaction between the NHS group with the antibody’s primary amine group can be noted in the anti-THC spectrum. The C-O bonds of the NHS ester are broken and react with primary amines of the antibody, resulting in a stable amide bond^26^. A suppression of peaks associated with the NHS ester (1799 cm^−1^, 1724 cm^−1^, 1251 cm^−1^) is observed, while peaks at ~1644 cm^−1^ and ~1552 cm^−1^ are indicative of amide I and amide II peaks. These results show a stable formation of the immunoassay through the binding of the anti-THC antibodies to the DSP functionalized surface.

### Analysis of zeta potential as a function of pH for the THC-BSA hapten

**Figure 3:**
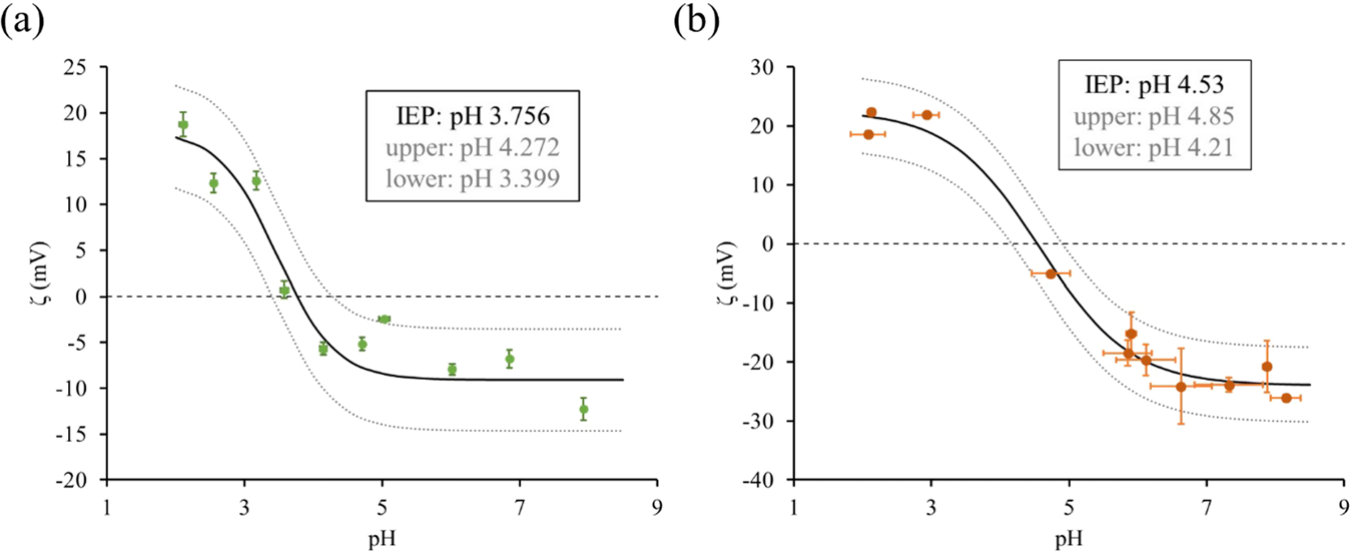
Zeta potential measurements in synthetic saliva at various pH for (**a**) the THC-BSA hapten and (**b**) BSA with no conjugation.

A sigmoid curve was fit to the experimental Zeta potential values using the nonlinear regression model in GraphPad Prism (Fig. 3). For the THC-BSA hapten the resulting sigmoid curve had an R-squared value of 0.9288, from which the isoelectric point was interpolated to be 3.756. An upper and lower 95% confidence interval was established for the sigmoid curve to estimate the bounds of the IEP which were 4.27 and 3.40. The sigmoid curve can be divided into three regions: steady positive zeta potentials, steady negative zeta potentials, and a transition phase. THC-BSA demonstrated positive zeta potentials (18.73 mV to 12.60 mV) at below pH 3, and negative zeta potentials (−5.67 mV to −12.26 mV) above pH 6. The transition phase of THC-BSA is between pH 3–5. The pH 3–5 phase is also the pH region in which the molecule is most unstable as its charge is closest to 0 mV.

To decouple the electrochemical nature of the hapten molecule, Zeta potential measurements were also taken under identical conditions with BSA proteins unconjugated to the THC biomarker. For the Zeta potential measurements on the BSA protein (no THC conjugation) a similar sigmoid curve was fit (R-squared value of 0.9785), to calculate its IEP of 4.53 with an 95% confidence interval bounds 4.85 and 4.21 (Fig. 3). Literature reports the theoretical and experimental IEP values for BSA to be around 4.7 and 4.6 respectively^29^.

Very little overlap exists in the 95% confidence intervals for the extrapolated IEP values for THC-BSA and BSA, thus it is likely that the presence of the THC dominates the electrochemical signature of the THC-BSA hapten. This finding supports that the EIS signal response measured using the electrochemical biosensor is driven by the presence of the THC molecule and not the BSA. Additionally, the Zeta potential for the THC-BSA hapten shows a relatively stable response within the normal pH range of human saliva (6.2–7.6), indicating that the THC-BSA charge and stability should be relatively stable across samples.

### Analysis of dose-dependent response via electrochemical impedance spectroscopy

**Figure 4:**
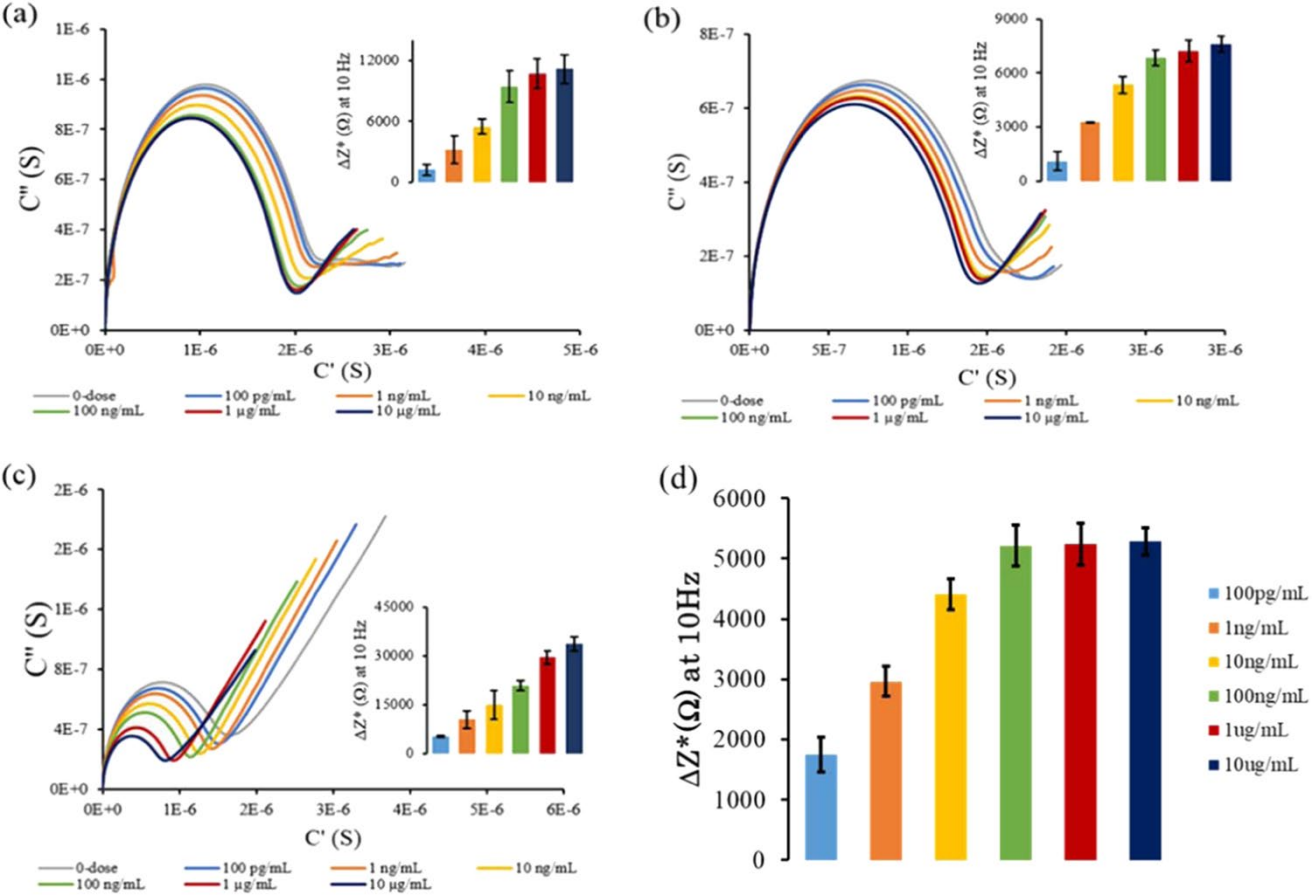
Calibrated dose response represented as Nyquist complex impedance plots with calibrated dose response inset for (**a**) 1x PBS (**b**) synthetic saliva at pH of 6 (**c**) synthetic saliva at pH of 4 (**d**) human saliva. All inserts represent the change in imaginary impedance (with respect to the 0-dose step) extracted at 10 Hz.

The electrochemical response due to binding events of the THC-BSA were reported as Nyquist capacitance plots (Fig. 4a–c) to resolve SAM dipolar characteristics and ionic permittivity. For experiments conducted in 1x PBS, the Nyquist capacitance plots exhibits a higher frequency semicircle with a less pronounced, low-frequency semicircle emerging with increasing concentrations of the THC-BSA hapten. Each of the semicircles corresponds to a time constant indicating that the immunoassay experiences two relaxation events. As the THC-BSA biomarker is introduced onto the sensor surface, a consequent decrease in the large semicircle diameter is observed. The decrease in semicircle diameter corresponds to a change in the Debye relaxation process due to a lengthening of the immunoassay as well as hindrance of ionic ingress toward the electrode surface. As the concentration of THC-BSA bound to the surface increases, rotational hinderance increases, reducing the size and number of pin holes in the immunoassay. Impedance values were extracted at 10 Hz and normalized across replicates (n = 4) by subtracting the ${Z}^{\ast }$ of the 0-dose step from each dose step (Fig. 4a inset). As higher doses are administered onto the sensor, an increase in the impedance response is observed. A similar trend is observed for the dose response experiment conducted in synthetic saliva at a pH of 6 (Fig. 3b). As the hapten binds to the immunoassay a similar decrease in the high frequency semicircle diameter is observed with similar manifestation of a second low-frequency semicircle. When plotting the dose response at 10 Hz after normalizing to the 0-dose step (subtracting the ${Z}^{\ast }$ of the 0-dose step from each dose step), the same trend was observed as the responses in 1x PBS and synthetic saliva at a pH of 6. This indicates that despite different capacitive processes dominating at the interface, the sensor can reliably detect varying doses of the THC-BSA hapten.

At a pH of 4 the dose response in the synthetic saliva exhibited slightly different interfacial characteristics as seen in Fig. 4c. The Nyquist capacitance plots display a similar high frequency semicircle corresponding to the Debye relaxation of the immunoassay. However, the low-frequency response is dominated by the diffusion of ions to the electrode surface as indicated by the 45° diffusion tail. This could in part, be due to the surface charges of the THC-BSA hapten at pH values near it’s IEP. The hapten exhibited only a weak surface charge at a pH of 4 ($\zeta \approx -\,5\,mV$), reducing the effect of the second Debye relaxation process and allowing the ionic diffusion to dominate the low-frequency response. However, a decrease in the higher frequency semicircle diameter is still observed, indicating that binding events of the THC-BSA hapten still play a role in the Debye relaxation process. While this pH lies outside of the normal salivary pH range, and thus not indicative of a normal sample, it is important to note that large fluctuations in saliva pH can occur after eating, drinking, or in response to infection.

The dose response of THC in commercially obtained human saliva (pH 6.0–7.0) at 10 Hz (n = 6 inter and intra assay replicates), as shown in Fig. 4d, shows an increasing trend in imaginary impedance change from the 0-dose step with increasing concentrations which can be correlated to the results obtained for synthetic sweat pH 4 and 6. At lower dose concentrations between 100 pg/ml – 100 ng/ml, the impedance changes are significantly distinguishable from each other. At higher dose concentrations of 1 ug/ml and 10 ug/ml, subtle changes in impedance from the baseline are observed and can be attributed to charge screening effects hindering the THC from being detected in a highly ionic human buffer. However, it is imperative to be able to detect THC in the lower regimes i.e. 4 ng/mL which is set as the oral fluid drug screening cut-off limit as per SAMSHA guidelines.

### Optimal operational frequency selection via FCBF

To select the most predictive feature(s), FCBF the symmetrical uncertainty (SU) was used to determine the information gained by including an individual feature. The FCBF algorithm was also slightly modified to include the 5 best features (FCBFK) to view other high performing features. Both a simple decision tree^19^ classifier and a logistic regression classifier were employed to test the performance before and after applying the FCBF algorithm. Decision trees are classifiers that search for the best location to split the data into pure groups given a random subset of features and inputs. Decision tree algorithms are a good candidate for FCBF validation as the classification accuracy should improve when selecting features with the highest information gain. The predictive performance for both the decision tree and logistic regression algorithms with and without the FCBF(K) algorithms were evaluated and summarized in Table 1.

**Table 1:** Classification accuracy of both Decision Tree and Logistic Regression on dataset with no feature selection and feature selection using FCBF and FCBFK algorithms.

	Raw Impedance Data (no normalization)		Normalized to Synthetic Saliva Measurement		Normalized to Antibody Measurement	
	Decision Tree	Logistic Regression	Decision Tree	Logistic Regression	Decision Tree	Logistic Regression
No Feature Selection	0.710	0.713	0.746	0.721	0.765	0.724
FCBF	0.901	0.845	0.824	0.813	0.699	0.632
FCBFK	0.820	0.702	0.652	0.813	0.742	0.746

The FCBF algorithm was evaluated separately first on the raw dataset, then on the dataset containing the impedance values normalized to the antibody step, and finally on the dataset containing impedance values normalized to the synthetic saliva step to select the most predictive feature from each. By removing noisy or correlated variables, FCBF selects a best performing feature with the goal of improving both the Decision Tree and Logistic Regression classifier accuracy. The FCBF algorithm improved the performance on both the raw impedance data and the data normalized to the synthetic saliva measurement by selecting a single feature from each of the sets: ${Z}^{\ast }$ at 398.0 Hz and $\% d{Z}_{\varphi }$ at 198.6 Hz – where ${Z}^{\ast }$ represents the raw impedance modulus and $\% d{Z}_{\varphi }$ represents the percent change in phase with respect to the synthetic saliva measurement.

**Figure 5:**
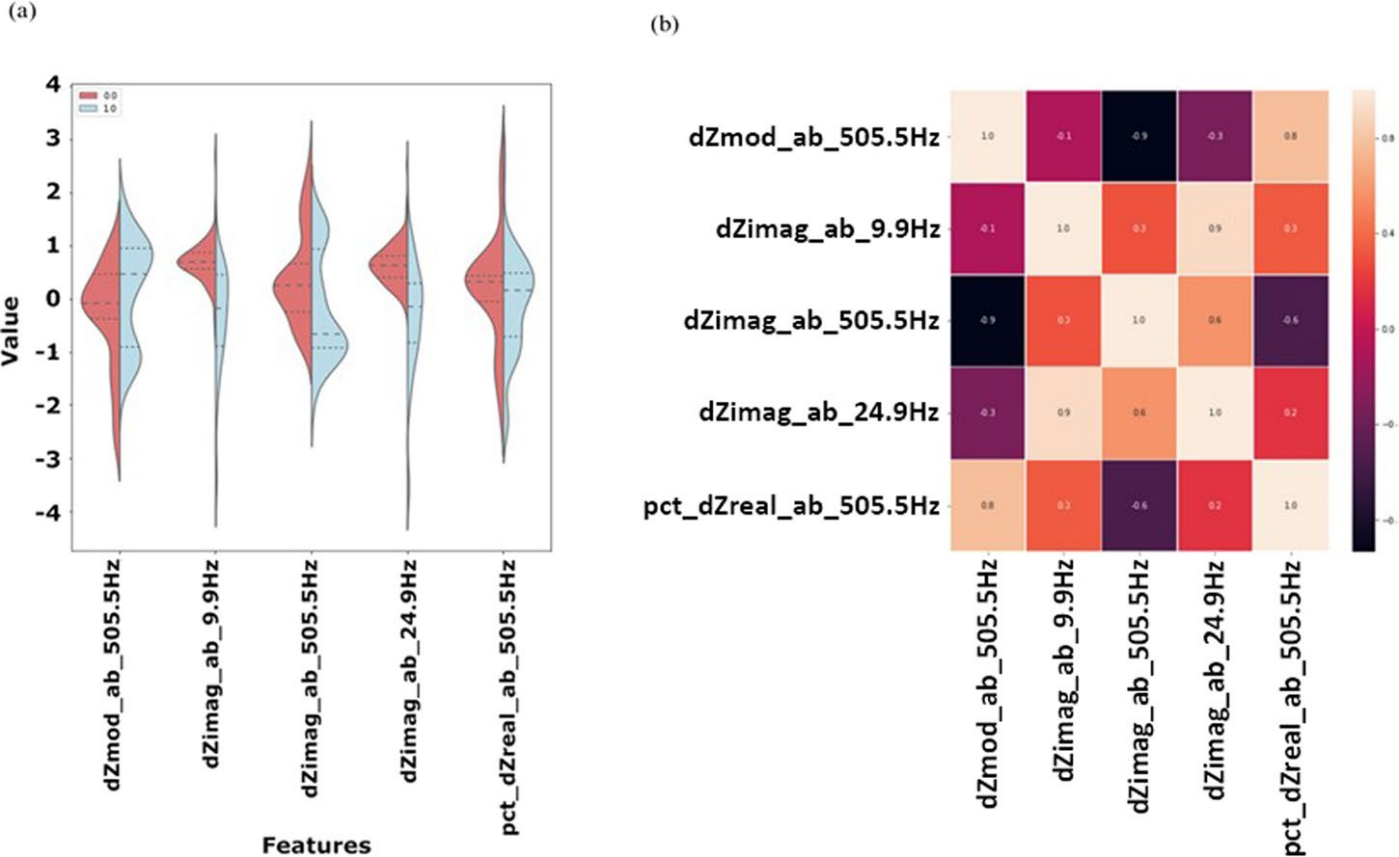
(**a**) Violin plots displaying normalized distributions and (**b**) correlation heatmap displaying Pearson’s correlation of the 5 features identified as most predictive by the FCBFK algorithm for the normalized to the antibody step.

However, the performance on the data normalized to the antibody measurement did not improve after reducing the feature space to a single feature. Results of the 5 best features from this dataset are shown in Fig. 5. The $d{Z}^{\ast }$ (change in impedance modulus with respect to the antibody measurement) at 505.5 Hz shows a bimodal distribution for the $THC+$ class with the mean of the $THC-$ class falling between both modes. This distribution of the data would greatly increase the complexity of the algorithm, so the $dZ\mbox{''}$ (change in imaginary impedance with respect to the antibody measurement) at 9.9 Hz was selected as the best feature for separating the data by class ($THC\,+\,/-$).

**Figure 6:**
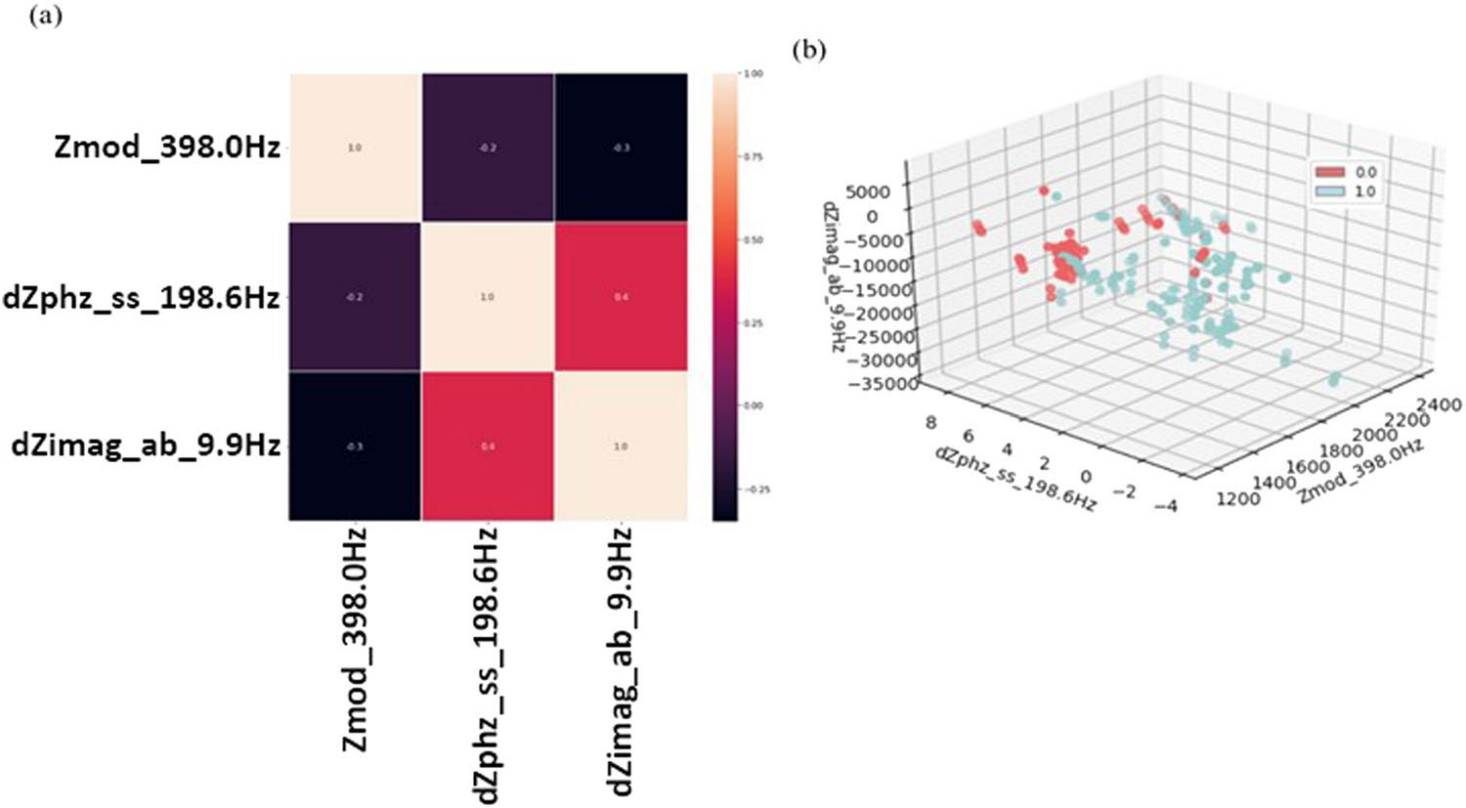
(**a**) Correlation heatmap and (**b**) 3D scatter-plot of final three selected features after the FCBF & FCBFK algorithms.

The final three features selected after implementing the FCBF(K) algorithm were ${Z}^{\ast }$ at 398.0 Hz, $\% d{Z}_{\varphi }$ (wrt synthetic saliva) at 198.6 Hz, and $dZ\mbox{''}$ (wrt antibody) at 9.9 Hz. Figure 6a shows that none of these features are correlated to one another while Fig. 6b demonstrates the dispersion of the data points in the reduced feature space.

### Binary Classification of *THC*+/− via Logistic Regression

Two Logistic Regression classifiers were constructed to make predictions on the data. The first logistic regression classifier was trained on a random subsample consisting of 80% of the input dataset with the remaining 20% held-out for model validation. The second logistic regression model implements cross-validation by partitioning the data into 10 equal bins using K-Folds cross-validation. The performance of each Logistic Regression model was evaluated on the data stored in the test dataset. Samples utilized for the binary classification studies were obtained from human subjects as per the IRB and then spiked with THC. Summaries of both the Logistic Regression and SVM classifiers are outlined below in Table 2.

**Table 2:** Performance summary of Binary Classification Algorithms.

Classifier Name	AUC	Threshold	TPR	FPR
Logistic Regression	0.911	0.39	97.1	60.0
		0.722	88.6	15.0
Logistic Regression with K-folds cross-validation	0.916	0.303	97.1	60.0
		0.769	88.6	10.0
Linear kernel SVM	0.911	0.214	97.1	60.0
		0.829	88.6	15.0
Radial Bias Function kernel SVM	0.951	0.621	97.1	15.0
		0.797	0.943	5.0

When performing Logistic Regression without K-folds cross-validation the AUC was 0.911. This high AUC points toward relatively good generalizability, and at a classification threshold of 0.39 the TPR of the model is 97.1%. However, at this threshold the FPR was 60% indicating that the majority of $THC-$ samples were being misclassified. A threshold of 0.722 reduced the FPR to 15%. However, this comes as a trade-off when classifying the $THC+$ samples; increasing the threshold decreases the TPR to 88.6%.

The AUC was 0.916 for the Logistic Regression model using K-folds cross-validation. At a classification threshold of 0.303, again the model had a TPR of 97.1% and a FPR of 60%. By increasing the threshold to 0.769, the TPR and FPR decreased to 88.6% and 10% respectively. This indicates that using K-folds cross-validation, the model had slightly better performance at predicting the $THC+$ samples, however the marginal increase in AUC demonstrates that the general performance was not significantly improved.

### Binary Classification of *THC*+/− via Support Vector Machines

In each of the SVM models, the kernel type and the regularization parameter C were passed as additional inputs for the SVM algorithm (gamma was also specified for the rbf SVM). For the linear kernel SVM a regularization parameter of C = 1 was set to reduce the impact of outlier data points when fitting the hyperplane. Other values of C were explored, however there were no significant changes in the model performance. The rbf kernel SVM used a C value of 100. While this reduces the margin size, a low value for the gamma parameter (set to 10) was used to discourage overfitting by increasing the radius of influence of the support vectors.

**Figure 7:**
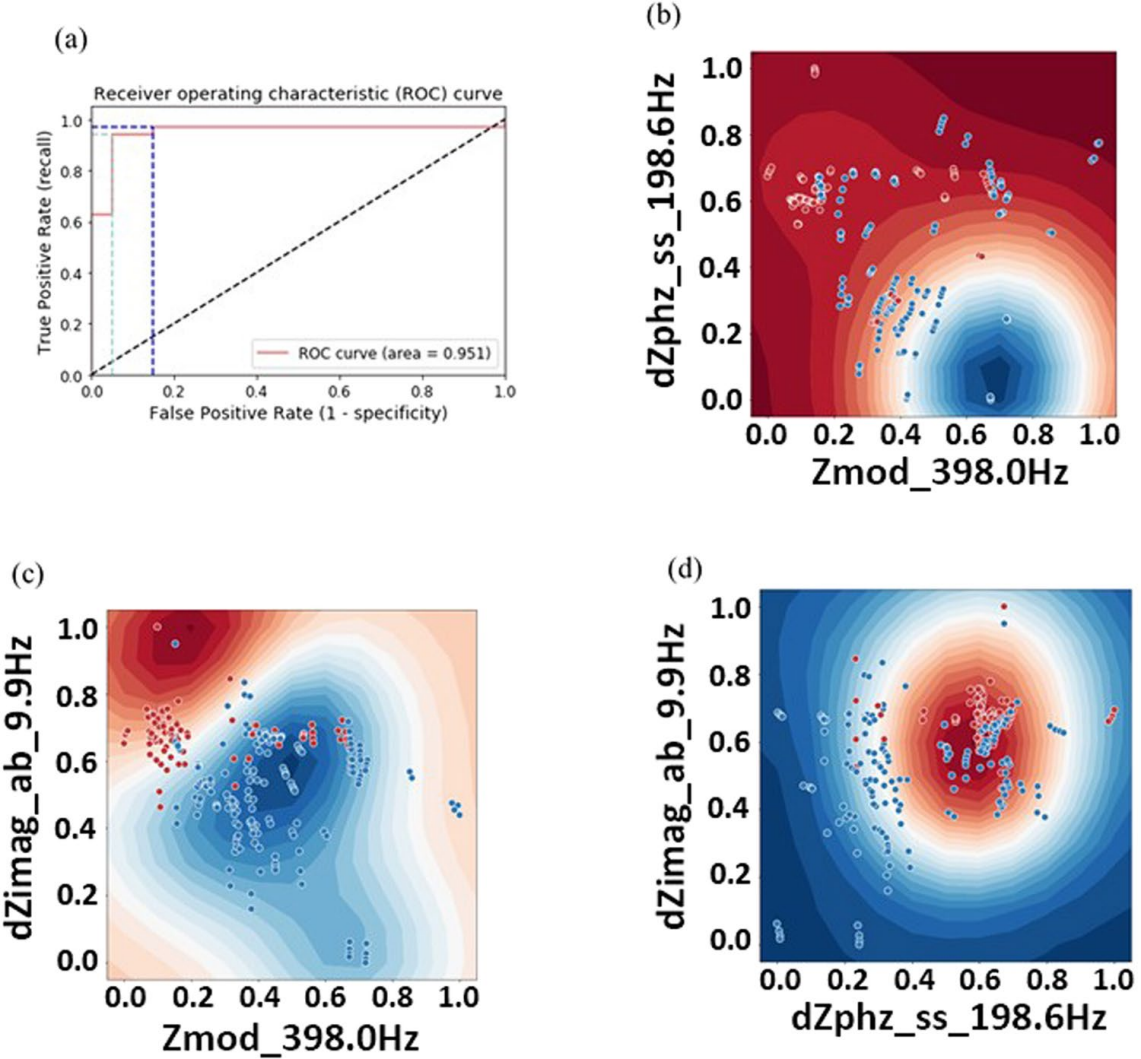
Rbf kernel SVM classification performance metrics. (**a**) ROC curve with TPR and FPR identified at thresholds 0.621 (dark blue) and 0.797 (light blue). (**b**–**d**) 2D cross-sections of the classification probability.

The linear kernel SVM was nearly identical to the first Logistic Regression model. Both models had similar ROCs with equivalent AUCs. This does not come as a large surprise, as SVM uses a linear approximation of the Logistic Regression cost function. Both models showed similar TPR and FPR (at slightly different cut-off thresholds), however a linear kernel SVM did not perform better than Logistic Regression with K-folds cross-validation. The rbf kernel SVM had the highest overall model performance and generalizability. As seen in Fig. 7b–d, most of the $THC-$ data points are clustered in a spherical region. Non-linear decision boundaries better grouped the two classes as indicted by the AUC (0.951). Furthermore, at a threshold of 0.621 the TPR and FPR for the rbf SVM were 97.1% and 15% respectively. The FPR reduced to 5.0% by increasing the threshold to 0.797 while only reducing the TPR to 94.3%.

## Discussion

We have developed a biosensor capable of detecting THC spiked within human saliva samples. This work utilizes non-faradaic EIS to rapidly and precisely detect the THC-BSA hapten with high specificity and sensitivity across numerous patients and patient samples. Additionally, this work demonstrates a rapid, unbiased procedure for determining the optimal operating frequencies using the FCBF algorithm. Reducing the input dimensionality and identifying the key measurements for future implementation of the salivary THC sensor allow for shorter measurement times and enhanced generalizability by reducing overfitting to noise within the dataset.

This work outlines a biosensor capable of detecting THC within saliva, independent of the sample-to-sample variability. Implementing a non-linear rbf kernel SVM algorithm to perform binary classification demonstrated a very high general performance (AUC = 0.951) at predicting the presence of THC within human saliva. This technology demonstrates a proof-of-concept for a roadside screening device for identifying drivers under the influence of marijuana. The sensor generates a rapid measurement of THC within a 1 minute with high specificity and sensitivity which are a necessity for law enforcement when administering roadside tests for marijuana impairment. The findings of this work can be translated to a portable, battery-powered device interfaced with a one-time use saliva swab embedded with the THC detection sensor for easier roadside testing when implemented using low-power EIS hardware^30–35^. While the work presented here focused on binary classification to identify the presence of THC, different learning algorithms could be explored via a regression analysis after FCBF(K) to predict concentrations of THC in saliva. A regression analysis could help identify a more accurate measurement of THC’s intoxicating effects and establish more precise cut-off limits for activities like driving under the influence. Additionally, the compact sensor design can be modified slightly to include multiplexed detection by simply swapping the anti-THC antibody for an antibody specific to THC-COOH, a THC metabolite, to build a more sophisticated model for predicting the time of most recent consumption.

## Data Availability

All data generated or analyzed during this study are included in this published article.
